# Molecular evolution of Cide family proteins: Novel domain formation in early vertebrates and the subsequent divergence

**DOI:** 10.1186/1471-2148-8-159

**Published:** 2008-05-23

**Authors:** Congyang Wu, Yinxin Zhang, Zhirong Sun, Peng Li

**Affiliations:** 1 Protein science laboratory of Ministry of Education, Department of Biological Sciences and Biotechnology, Tsinghua University, Beijing 100084, China; 2 MOE Key Laboratory of Bioinformatics, State Key Laboratory of Biomembrane and Membrane Biotechnology, Department of Biological Sciences and Biotechnology, Tsinghua University, Beijing 100084, China

## Abstract

**Background:**

Cide family proteins including Cidea, Cideb and Cidec/Fsp27, contain an N-terminal CIDE-N domain that shares sequence similarity to the N-terminal CAD domain (NCD) of DNA fragmentation factors Dffa/Dff45/ICAD and Dffb/Dff40/CAD, and a unique C-terminal CIDE-C domain. We have previously shown that Cide proteins are newly emerged regulators closely associated with the development of metabolic diseases such as obesity, diabetes and liver steatosis. They modulate many metabolic processes such as lipolysis, thermogenesis and TAG storage in brown adipose tissue (BAT) and white adipose tissue (WAT), as well as fatty acid oxidation and lipogenesis in the liver.

**Results:**

To understand the evolutionary process of Cide proteins and provide insight into the role of Cide proteins as potential metabolic regulators in various species, we searched various databases and performed comparative genomic analysis to study the sequence conservation, genomic structure, and phylogenetic tree of the CIDE-N and CIDE-C domains of Cide proteins. As a result, we identified signature sequences for the N-terminal region of Dffa, Dffb and Cide proteins and CIDE-C domain of Cide proteins, and observed that sequences homologous to CIDE-N domain displays a wide phylogenetic distribution in species ranging from lower organisms such as hydra (*Hydra vulgaris*) and sea anemone (*Nematostella vectensis*) to mammals, whereas the CIDE-C domain exists only in vertebrates. Further analysis of their genomic structures showed that although evolution of the ancestral CIDE-N domain had undergone different intron insertions to various positions in the domain among invertebrates, the genomic structure of *Cide *family in vertebrates is stable with conserved intron phase.

**Conclusion:**

Based on our analysis, we speculate that in early vertebrates CIDE-N domain was evolved from the duplication of NCD of Dffa. The CIDE-N domain somehow acquired the CIDE-C domain that was formed around the same time, subsequently generating the Cide protein. Subsequent duplication and evolution have led to the formation of different Cide family proteins that play unique roles in the control of metabolic pathways in different tissues.

## Background

Cide family proteins including Cidea, Cideb and Cidec/Fsp27 [1-3] were originally identified by their sequence homology to the N-terminal CAD domain (NCD) [4] of DNA fragmentation factors Dffa and Dffb [5-16]. Whereas NCD specifically refers to the N-terminal domain of Dff factors, CIDE-N denotes the N-terminal sequence shared by Cide proteins in this article. In addition to the CIDE-N domain, Cide proteins also contain a unique conserved C-terminal domain (CIDE-C domain). Despite some variation between NCD and CIDE-N, they all contain a potential yin and yang surface that could mediate weak protein-protein interaction. Recently, they were also found to be structurally homologous to the ubiquitin (UB) α/β roll superfold [17,18], but bear no high similarity to other existing proteins [17,19].

While *Cidea *is expressed at high levels in BAT [20], *Cideb *is more abundantly expressed in the liver, with moderate levels in kidney, small intestine and colon [21]. When over-expressed in heterologous cells such as 293T and COS-7 cells, Cideb can form homo- or hetero-dimers with other CIDE family members and induce caspase-independent cell death [22]. Furthermore, CIDE-C domain of Cideb, is responsible for Cideb-induced cell death and dimerization [22]. Cidea protein has also been found to regulate apoptosis induced by TGF-β [23]. However, how Cide proteins induce apoptosis remains unclear. No caspase cleavage site or nuclease specific domain present in Dff factors was identified in Cide proteins. To study the physiological role of Cide proteins, we previously generated *Cidea *null mice, and found that *Cidea*-null mice are lean and resistant to diet-induced obesity and diabetes [20]. Cidea controls energy homeostasis in BAT by regulating lipolysis and thermogenesis. A recent study showed that *Cidea *was implicated in human obesity by regulating human adipocyte lipolysis [24] and a V115F polymorphism in human was found to be associated with obesity in certain populations [25]. *Cidea *was the most highly up-regulated gene in the liver of high calorie diet (HC)-fed mice and second most down-regulated gene in the liver of HC plus resveratrol (HCR) aging-improved mice [26]. Similar to Cidea, Cideb also plays important roles in metabolism. We recently reported that *Cideb *regulates diet-induced obesity, liver steatosis, and insulin sensitivity by controlling lipogenesis and fatty acid oxidation in the liver [21]. In addition, Fsp 27/Cidec was found to be associated with lipid droplets and promote triglyceride storage in differentiated 3T3-L1 cells [27,28]. All these studies suggest that Cide family proteins play important roles in modulating energy homeostasis, aging and the development of metabolic diseases such as obesity and diabetes [29-31].

While it is evident that Cide proteins regulate energy homeostasis in mammals, it is unclear about the origin and evolution of Cide family proteins. To provide further insights into the structure and function of Cide proteins, we have employed various databases and bioinformatic tools to study how Cide family proteins have been evolved. A recent analysis of the evolutionary process of Dff family proteins has identified orthologs of Dffa/b in lower organisms such as sea anemone, suggesting that the DNA fragmentation pathway in apoptosis is conserved throughout evolution [32]. Here we defined signature sequences for the N-terminal region of Dffa, Dffb and Cide proteins and CIDE-C domain of Cide proteins and analyzed the evolutionary history of CIDE-N and CIDE-C domains of Cide family proteins. No ortholog of CIDE proteins was identified in invertebrates or other lower organisms. However, a homologous sequence of CIDE-N domain of Cide proteins was identified in hydra, in addition to sea anemone as previously reported [32]. We found that the signature sequences for CIDE-N domain of Cide proteins are similar to those of NCD of Dffa in hydra and sea anemone. More importantly, we found that CIDE-C domain exists only in vertebrates with occasional possible omission from certain ancient fish species. By analyzing the genic structures and intron phases of Cide and Dff family, we found that although the evolution of the ancestral CIDE-N domain includes different intron insertions, the genomic structure of *Cide *family in vertebrates is stable, including 5 conserved exons separated by 4 introns with the sequential phases 2-0-0-2. Based on our observation, we postulate that the origin of Cide proteins may be the result of recombination of sequences encoding CIDE-N and CIDE-C domains in early vertebrates, and subsequent duplication and evolution have led to the formation of different Cide family proteins.

## Results

### Sequence comparison and species/tissue distribution of CIDE-N and CIDE-C domains

**Figure 1:**
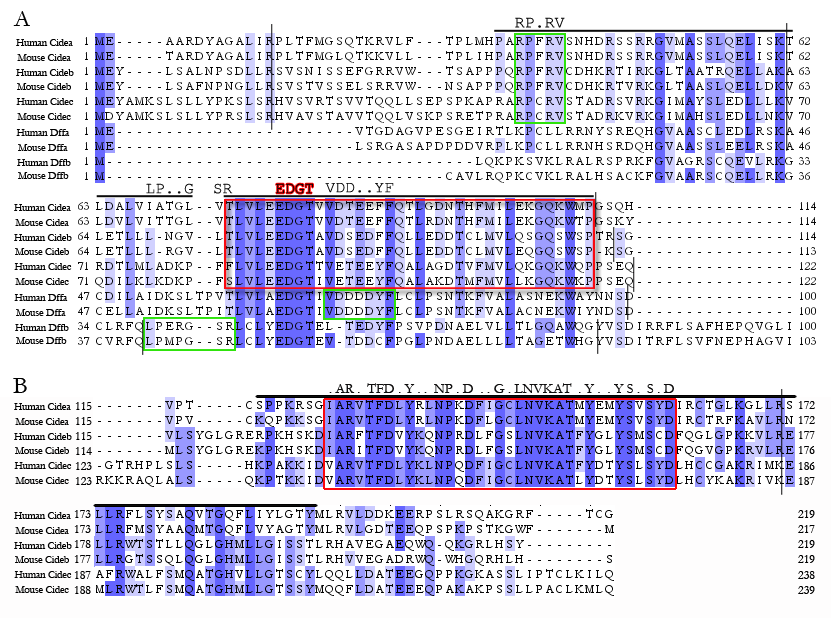
**The analysis of Cide and Dff family proteins in human and mouse**. (A) Sequence alignment of the N-terminals of all available Cide and Dff family proteins in human and mouse using MAFFT algorithm. The CIDE-N domain is indicated by a dark line on top of the alignment. The alignment of the most conserved region of 37 amino acids encompassing the EDGT signature motif is framed with a red rectangle. The signatures of Cide and Dff family proteins are framed with a green rectangle. The exon boundaries are marked by black vertical lines. (B) Sequence alignment of the CIDE-C domains of Cide family proteins found in human and mouse using MAFFT algorithm. The CIDE-C domain is indicated by a dark line on top of the alignment. The alignment of most conserved 35 amino acids is framed with a red rectangle. The exon boundaries are marked by black vertical lines.

Through sequence alignment of the N-terminal region of Cide and Dff family proteins in human and mouse (Fig 1a), we observed highly conserved 37 amino acid residues around the EDGT protein signature site in the CIDE-N domain and NCD. NMR structural analysis in human Cideb suggests that the EDGT signature is located on an important loop of the CIDE-N domain interaction interface zone 1. Within this conserved domain, we observed penta amino acid residues RPXRV unique for CIDE-N domain of Cide family proteins, a VDDXXYF signature for Dffa and a LPXXGSR signature for Dffb (Fig 1a). These specific sequences will be used to distinguish Cide proteins from Dff in our following study. In addition, through the sequence alignment of CIDE-C domain of Cide family proteins in human and mouse (Fig 1b), we identified a highly conserved XARXTFDXYXXNPXDXXGXLNKVATXYXXYSXSXD signature in CIDE-C domain.

An Hmmer search of Nr.db downloaded from NCBI showed that proteins that share similarity to CIDE-N domain or NCD are found in 25 organisms (Table 1), representing either CIDE or Dff proteins. These Dff proteins are widely found in vertebrates and invertebrates, whereas Cide proteins only exist in 16 vertebrates among the 25 organisms.

**Table 1:** Hmmer search in Nr.db from NCBI using the CIDE-N motif pfam02017

Organism/Protein Found	Cidea	Cideb	Cidec	Dffa Related	Dffb Related	Drep1* Related	Drep2* Related	Drep3* Related	Drep4* Related
*Homo sapiens *(human)	1	1	1	1	1				
*Pan troglodytes*	1		1	1	1				
*Pongo pygmaeus*				1					
*Macaca mulatta*	1		1	1	1				
*Mus musculus *(mouse)	1	1	1	1	1				
*Rattus norvegicus*	1	1	1	1	1				
*Equus caballus*	1	1	1	1					
*Bos Taurus*	1	1	1	1	1				
*Sus scrofa*	1	1	1						
*Canis lupus familiaris*	1	1	1	1	1				
*Monodelphis domestica*		1	1	1	1				
*Ornithorhynchus anatinus*	1		1	1	1				
*Gallus gallus *(chicken)	1			1	1				
*Xenopus laevis*	1	1	1						
*Xenopus tropicalis *(*X.tropicalis*)	1	1	1	1					
*Danio rerio *(zebrafish)	1	1	1	1	1				
*Tetraodon nigroviridis *(tetraodon)		1	1	1	1				
*Aedes aegypti*						1	1		1
*Anopheles gambiae str. PEST*							1		1
*Culex pipiens quinquefasciatus*						1	1		1
*Drosophila melanogaster *(fruit fly)						1	1	1	1
*Drosophila pseudoobscura*						1	1	1	1
*Bombyx mori*						1			
*Apis mellifera*						1	1		
*Tribolium castaneum*							1		1

Total Cide Protein Found 41

Total Dff Related Protein Found 48

* Drep1 is Dffa in fruit fly, and Drep4 is Dffb in fruit fly.

To check the expression of Cide proteins, we searched currently available EST data base using the mouse CIDE-N sequence and found 1,251 EST clones that share homology to CIDE-N domain or NCD in 71 organisms (Table 2) spanning from lower organism such as cnidarians (*H. vulgaris *and *N. vectensis*) to human. These data suggest that CIDE-N domain is evolutionarily conserved, consistent with an observation previously reported [32]. Some 857 EST clones that share sequence homology to the CIDE-C domain were found in 37 organisms (Table 2), all of which are vertebrates including sharks, bone fish, amphibians, birds, and mammals. Thus far, we have not identified any protein or open reading frame that contains only the CIDE-C domain and does not contain the CIDE-N domain. The Nr.db and EST search also suggests that CIDE-C domain has appeared in a later stage of evolution and it may have a specific function relating to vertebrates.

**Table 2:** EST distribution chart for organisms found with CIDE related domains

Organisms Found with Cide Related Domains	Species	Search Criterior		Tissue Distribution#		
		
		CIDE-N 37aa	CIDE-C 35aa	Cidea	Cideb	Cidec/Fsp27
*Homo sapiens *(human)	**Mammals**	281	126	BAT+, WAT, skin, heart, sympathetic trunk	liver, small intestine+, kidney, colon, lung, brain, thalamus, cervix, thymus, T-lymphocytes, lymph, placenta, stomach, prostate, testis, eye	WAT, white matter, brain, lung, skin, eye, breast, ovary, uterine, pancreas, colon, testis, nasopharynx
*Macaca fascicularis*		3	1			
*Macaca nemestrina*		1				
*Macaca mulatta*		1	1			
*Pongo pygmaeus*		3				
*Papio anubis*		2				
*Spermophilus lateralis*			1			
*Mus musculus *(mouse)		185	130	BAT+, mammary gland, salivary gland, aorta, thyroids, eye, head, lung, kidney	liver, kidney, colon, small intestine+, bowel, placenta	WAT+, mammary gland, salivary gland, thyroid, colon, lung, kidney, placenta
*Rattus norvegicus*		13	15			
*Rattus sp*.		1	1			
*Sus scrofa*		89	74			
*Bos taurus*		98	90			
*Ovis aries*		13	5			
*Capra hircus*		1				
*Canis lupus familiaris*		9	10			
*Macropus eugenii*		2	2			
*Trichosurus vulpecula*		11	9			

*Gallus gallus *(chicken)	**Birds**	40	36	ovary, brain, caecal tonsil, intestinal lymphocyte, liver		liver, small intestine, fat body, intestinal lymphocyte, hearts
*Taeniopygia guttata*		4	4			
*Meleagris gallopavo*		1				

*Xenopus tropicalis *(*X.tropicalis*)	**Amphibians**	149	146	ovary, brain	liver, gut/intestine, fat body, oviduct	Small intestine, lung brain
*Xenopus laevis*		88	77			
*Ambystoma mexicanum*		1	1			

*Danio rerio *(zebrafish)	**Bone Fishes**	56	16	eyes	liver, gut/intestine	liver
*Dicentrarchus labrax*		1	1			
*Cyprinus carpio*		1	1			
*Fundulus heteroclitus*		4	3			
*Gasterosteus aculeatus*		13	3			
*Gadus morhua*		4	1			
*Misgurnus anguillicaudatus*		1				
*Ictalurus furcatus*		1	1			
*Oncorhynchus mykiss*		30	30			
*Oncorhynchus nerka*		1	1			
*Oryzias latipes *(medaka)		49	44		liver	ovary
*Pimephales promelas*		3				
*Platichthys flesus*		1	1			
*Plecoglossus altivelis altivelis*		1	1			
*Salmo salar *(Atlantic salmon)		12	15	testis	gut/intestine, head kidney, thymus, pyloric caecum	thyroid, thymus, spleen, pyloric caecum
*Sparus aurata*		2	2			
*Takifugu rubripes *(fugu)		2	1			
*Thunnus thynnus*		2	1			
*Zosterisessor ophiocephalus*		1	1			

*Leucoraja erinacea *(little skate)	**Sharks & Rays**	8	2			Liver
*Squalus acanthias *(spiny dogfish)		3	3			

*Branchiostoma floridae *(amphioxus)	**Lancelets**	3				

*Drosophila melanogaster *(fruit fly)	**Insects**	8				
*Drosophila pseudoobscura*		2				
*Drosophila ananassae*		1				
*Drosophila erecta*		1				
*Aedes aegypti*		3				
*Anopheles gambiae*		1				
*Lutzomyia longipalpis*		1				
*Phlebotomus papatasi*		1				
*Bombyx mori*		3				
*Apis mellifera*		1				
*Acyrthosiphon pisum*		3				
*Aphis gossypii*		1				
*Heliconius erato/himera mixed*		1				
*Heliconius melpomene*		1				
*Tribolium castaneum*		3				
*Diabrotica virgifera virgifera*		1				

*Rhipicephalus appendiculatus*	**Arachnids**	1				
*Ixodes scapularis*		2				

*Daphnia pulex*	**Crustaceans**	1				
*Homarus americanus*		1				

*Lottia gigantea*	**Molluscs**	4				
*Aplysia californica*		2				
*Crassostrea gigas*		2				
*Mytilus californianus*		2				

*Capitella sp. I ECS-2004*	**Annelids**	3				

*Nematostella vectensis *(sea anemone)	**Cnidarians**	5				
*Hydra vulgaris *(hydra)		1				

**Total EST Hits**		**1251**	**857**			

# The order of tissues is based on the copy number of cDNAs encoding corresponding protein. The cDNAs from mixed tissues are ignored in counting.

+ The high expresion of Cide proteins in these tissues is verified by expriments, but not observed in the current EST database.

To further investigate the origin and function of different Cide proteins, we checked the tissue distribution of ESTs encoding Cide proteins in vertebrates (Table 2). In the mouse, *Cidea *is predominantly expressed in BAT, with small amounts of mRNA detected in heart, brain, skeletal muscle, lymph node, thymus, appendix and bone marrow [2,20]. The expression of *Cidec*/*Fsp27 *is more widespread, with high levels in WAT and moderate levels in BAT and skeletal muscle[3,20]. *Cideb *is more abundantly expressed in the liver, with moderate levels in kidney, small intestine and colon [21]. Results from the analysis of the distribution of Cide ESTs are in good agreement with the above observations, revealing that a large number of EST for *Cideb *found in the liver, and of *Cidec *in WAT. We also found Cide proteins are expressed at varying levels in many different tissues in the lower vertebrates. *Cidea *is expressed in the eye of zebrafish (*Danio rerio*), testis of Atlantic salmon (*Salmo salar*), ovary and brain of *X. tropicalis *and chicken (*Gallus gallus*), caecal tonsil, intestinal lymphocyte and liver of chicken; *Cideb *is expressed in the liver of zebrafish, medaka (*Oryzias latipes*) and *X. tropicalis*, gut/intestine of zebrafish, Atlantic salmon and *X. tropicalis*, in the thymus, head kidney, and pyloric caecum of Atlantic salmon, in fat body and oviduct of *X. tropicalis*; while *Cidec/Fsp27 *is expressed in the liver of little skate (*Leucoraja erinacea*), zebrafish and chicken, in the small intestine of *X. tropicalis *and chicken, in the ovary of medaka, in thyroid, thymus, spleen and pyloric caecum of Atlantic salmon, in the brain and lung of *X. tropicalis*, as well as in the fat body, intestinal lymphocyte and hearts of chicken.

### Identification of the ancestral CIDE-N domain in hydra

Hydra and sea anemone belong to the phylum cnidaria which is one of the earliest animal phyla [33,34]. We found 1 cDNA (GenBank: DY447116) in hydra and 5 cDNAs (GenBank: DV089654, GenBank: DV085979, GenBank: FC181163, GenBank: FC273871 and GenBank: FC274613) in sea anemone which encode proteins homologous to the most conserved 37 amino acids of CIDE-N domain from mouse Cideb. Further analysis revealed that the cDNA in hydra encode Dffa, while the other 5 cDNAs in sea anemone all encode Dffa, as observed by Eckhart et al [32].

**Figure 2:**
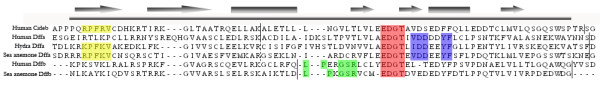
**Sequence alignment of N-terminals for Cide and Dff family proteins in hydra, sea anemone, and human**. Shown here are six N-terminals of Cide and Dff proteins, with the sequence from human Cideb representing the CIDE-N domain and the rest representing the NCD domains. The most conserved EDGT signature is highlighted in red. The RPXRV signature of Cide family proteins is highlighted in yellow, the VDDXXYF signature of Dffa in purple, and the LPXXGSR signature of Dffb in green. The exon boundaries are marked by black vertical lines. The potential exon boundaries are marked by black dotted lines. The secondary structure of the human Cideb's CIDE-N domain is presented on the top of the alignment [17]; cylinders and arrows represent α helices and β strands, respectively.

Sequence alignment of N-terminal region for Cide and Dff family proteins in hydra, sea anemone and human (Fig 2) showed a remarkable similarity between Cide proteins and hydra Dffa. Using pair-wise comparison, we found the NCD of hydra Dffa shares approximately 42.3 percent sequence similarity to the NCD of human Dffa, and 42.9 percent sequence similarity to the CIDE-N domain of human Cideb. Dffa in hydra and sea anemone has signatures of both Cide family proteins and Dffa (RPXRV and VDDXXYF), but Dffb in sea anemone only contain signature sequences for Dffb (LPXXGSR). These data, together with the above sequence comparison and species distribution data, suggest that CIDE-N domain of Cide proteins is derived from the NCD of Dffa, but not Dffb, in lower organism like hydra and sea anemone. Thus we define herein the NCDs of Dffa in hydra and sea anemone as the ancestral CIDE-N domain.

### Comparative genomic analysis of genic structures and intron phases of *Cide *and *Dff *gene family

**Figure 3:**
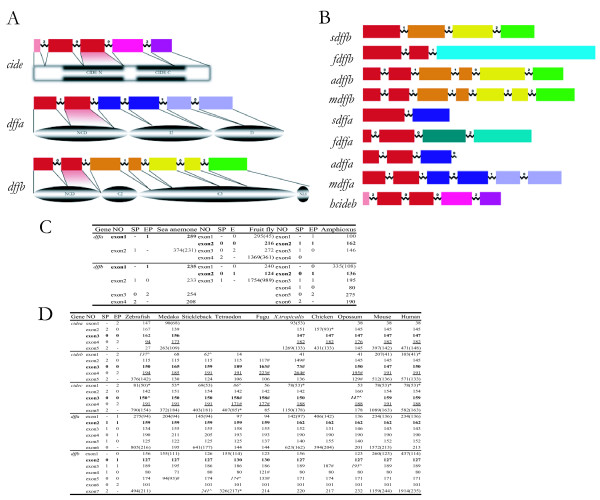
**Genic structures and intron phases of *Cide *and *Dff *gene family**. (A) The gene structures of human *Cideb*, mouse *Dffa *and mouse *Dffb *are shown above their corresponding proteins. Exons and introns are shown in boxes and wavy lines, respectively [4, 17]. Exons are drawn to scale. Black lines link a particular exon to its translated region of the protein. The matching areas flanked by black lines between the most conserved proteins sequences and their corresponding exons are makred in red. (B) Genic structures and intron phases of *Cide *and *Dff *gene family from several representative model organisms are drawn to scale. The regions from the various species in the same color share sequence homology. (C, D) The genic and intron phases of Cide and Dff gene family are divergent in invertebrates but conserved in vertebrates. Exon sizes in different organisms are shown in evolutional order. The bordering intron phases of the exons are shown in the left. The number in '()' of exon 1 of each gene indicates its nucleotide position downstream of ATG (inclusive of the three nucleotides). The number in '()' of each last exon indicates its nucleotide position upstream of its stop codon. '*' indicate before the first exon (or after the last exon) noncoding exon regions. '#' refers to exons with deviational start phase and the end phase from the others in the same exon group. '^' indicates discard of some flanking exons. Italicized numbers are adjusted exon sizes. Exons in bold encode the most conserved protein regions. Abbreviations for species: s, sea anemone (*Nematostella vectensis*); f, fruit fly (*Drosophila melanogaster*); a, amphioxus (*Branchiostoma floridae*); m, mouse (*Mus musculus*); h, human (*Homo sapiens*). Other model organisms are zebrafish (*Danio rerio*), medaka (*Oryzias latipes*), stickleback (*Gasterosteus aculeatus*), tetraodon (*Tetraodon nigroviridis*), fugu (*Takifugu rubripes*), *X.tropicalis *(*Xenopus tropicalis*), chicken (*Gallus gallus*), opossum (*Monodelphis domestica*). SP, start phase, EP, end phase.

By searching the genomic data base of various species, we observed that the gene structure of all Cide family proteins in vertebrates consists of 5 exons and 4 introns with the sequential phases 2-0-0-2, while vertebrate *Dffa *gene consists of 6 exons and 5 introns with the sequential phases 1-1-0-1-0. Vertebrate *Dffb *consists of 7 exons and 6 introns with the sequential phases 0-1-1-0-0-2. The length of exons of *Cide *gene family is also conserved in vertebrates. By matching their exons to the corresponding protein sequences of Cide and Dff family proteins, we found that CIDE-N domain is encoded by exon 2 and exon 3 of *Cide *genes whereas the conserved CIDE-C domain is encoded by exon 4 and exon 5 of *Cide *genes. NCDs of Dffa and Dffb are encoded mainly by its exon 1 and exon 2, respectively (Fig 3a,d).

Unlike the conserved genic structures and intron phases of *Dff *gene family in 10 vertebrates, the *Dff *gene family in 3 representative invertebrates has different genic structures and intron phases. The ancestral CIDE-N domain of Dffa in sea anemone was split by one phase 0 intron in fruit fly (*Drosophila melanogaster*), but by phase 1 intron in a different position in amphioxus (*Branchiostoma floridae*) (Fig 3b,c). Based on the genomic structure and analysis from non-redundant proteins databases and EST database, we conclude that the *Cide *gene family exists only in vertebrates, while the *dff *gene family exists in both vertebrates and invertebrates.

### The absence of some Cide family proteins in several vertebrate species

From the result of tblastn search in available EST data bases, we found the most ancient CIDE-N domain exists in hydra and sea anemone, and the most ancient CIDE-C domain exists in spiny dogfish (*Squalus acanthias*) and little skate (*Leucoraja erinacea*). No sequence homologous to CIDE-N domain was identified before phylum cnidaria such as yeast (*S. cerevisae*). In addition, no sequence homologous to CIDE-C was found before the phylum vertebrata.

**Figure 4:**
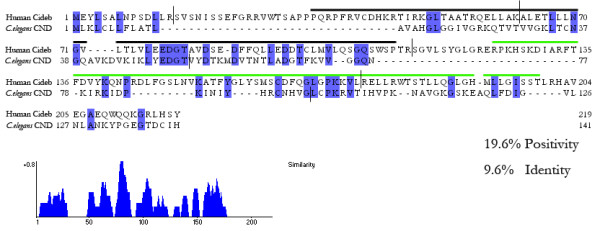
**The full sequence alignment of human Cideb and the putative CIDE-N domain-containing protein (CND) in *C. elegans***. The CIDE-N domain is marked by black bold line and the CIDE-C domain by green bold line. The exon boundaries are marked by solid vertical lines.

Interestingly, although the whole genome of nematodes (*C. elegans*) was sequenced and analyzed extensively, no genomic sequence encoding proteins that share sequence similarity to Dffa/b or Cide proteins were identified. We only found 1 protein (GenBank: Y51A2D.10) in *C. elegans *with limited homology to the conserved 37 amino acids including the signature EDGT motif of CIDE-N domain from human Cideb (Fig 4), with no homology to any other region. Furthermore, the exon boundaries between this protein and human Cideb are not conserved. Therefore, this protein is unlikely to be the ortholog of Dff or Cide in *C. elegans*.

In the Petromyzon_marinus-3.0 Contigs database (sea lamprey (*Petromyzon marinus*) genome data base last modified on Apr 16, 2007) we found an ortholog of Dffb, but not Dffa or Cide proteins. As sea lamprey is regarded as the most primitive vertebrate, it would be interesting to determine whether any ortholog of Cide proteins exists in this organism. In the large EST database for little skate and spiny dogfish, we failed to identify any Cidea-like protein. Thus it is highly possible that ortholog of Cidea may not exist in little skate and spiny dogfish. Furthermore, after searching the whole genome sequences of fugu (*Takifugu rubripes*) and tetraodon (*Tetraodon nigroviridis*) [35,36], no ortholog for Cidea protein was identified in these species. No Cidea-like protein was identified in stickleback (*Gasterosteus aculeatus*) either. In addition, no ortholog of Cideb was identified in the currently available chicken (*Gallus gallus*) genome data base [37] or EST databases.

### The phylogenetic tree of CIDE-N and CIDE-C domains

**Figure 5:**
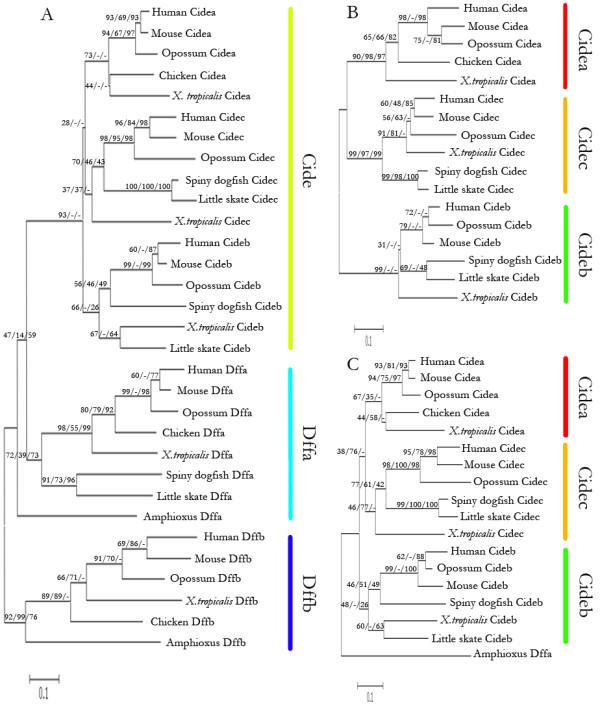
**Neighbor-joining phylogenetic trees of selected Cide or Dff family proteins from model organisms**. (A) Shown here is the NJ phylogeny of 31 representative Cide and Dff family proteins from various model organisms. The protein sequences conserved by CIDE and Dff family proteins were used and the tree was drawn by using MEGA 4.0. (B) The NJ phylogeny of 17 selected Cide family proteins in vertebrates based on the CIDE-C domain rooted by the NCD of amphioxus Dffa. (C) The NJ phylogeny of 17 selected Cide family proteins in vertebrates based on the CIDE-N domain rooted at midpoint. Bootstrap values for NJ, ML and UPGMA analyses (first, second and third values, respectively) are presented for each clade. The scale bar indicates the number of amino acid substitutions per site. Model organisms used are amphioxus (*Branchiostoma floridae*), little skate (*Leucoraja erinacea*), spiny dogfish (*Squalus acanthias*), *X.tropicalis*, chicken (*Gallus gallus*), opossum (*Monodelphis domestica*), mouse (*Mus musculus*), human (*Homo sapiens*).

In this study, three methods including neighbor-joining (NJ), maximum likelihood (ML), and unweighted pair group method with arithmetic mean (UPGMA) were used to construct the phylogenetic trees. These three methods often gave the same trees, except for some minor details. From the phylogeny of 31 selected Cide and Dff family proteins from various model organisms using the CIDE-N domain and NCD, respectively (Fig 5a), we found that Cide family proteins form an independent sub-clade from the Dffa proteins and the vertebrate CIDE-N domains have close relationship with the NCD of amphioxus Dffa. These results confirm that CIDE-N domain is derived from NCD of Dffa in early vertebrates. Through the two phylogenetic trees of 17 selected Cide family proteins in vertebrates using the CIDE-N and CIDE-C domains, respectively (Fig 5b,c), we found that all the Cide family proteins can be divided into 3 subfamilies, Cidea, Cideb and Fsp27/Cidec. The CIDE-N domain NJ phylogeny is rooted by the NCD of amphioxus Dffa (Fig 5a,c). In addition, the CIDE-N domain ML phylogeny and the CIDE-C domain phylogeny generated by NJ and UPGMA analysis are rooted at midpoint (Fig 5b). These data suggest that Cideb is the most ancient member in Cide family, and its duplication resulted in the formation of Cidec and Cidea. However, the CIDE-N domain UPGMA phylogeny rooted by the NCD of amphioxus Dffa indicates Cidec as the most ancestral Cide protein. The CIDE-C domain ML phylogeny rooted at midpoint indicates Cidea as the most ancestral Cide protein. To sum up, these results point to a likelihood that Cideb is the most ancestral Cide protein.

## Discussion

Here we have defined signature sequences for the N-terminal region of Dffa, Dffb and Cide proteins, CIDE-C domain of Cide proteins, and analyzed the evolutionary history of CIDE-N and CIDE-C domains of Cide proteins using various databases and bioinformatic tools. We identified the ancestral CIDE-N domain in hydra and found CIDE-C domain exists only in vertebrates. Furthermore, genomic structures and intron phases of Cide family proteins are conserved in vertebrates.

### The formation of ancestral CIDE-N in early metazoan

Based on currently available data, we have found the ancestral CIDE-N domain in *H. vulgaris*, in addition to *N. vectensis *as previously reported [32]. In tropical demosponge *Reniera*, more primitive to the above two cnidarians, we did not find any sequence homologous to CIDE-N domain, with the caution that its genome sequencing is still ongoing [38]. Similarly, we did not find any CIDE-N homology in the current database of EST from sponges [39]. However, other important apoptosis genes, such as the proapoptotic molecule DD2 [40], the cell survival proteins, Bcl-2-related molecules [40], and caspases [41] have been found in the sponge. More importantly, the caspase-3 dependent DNA fragmentation was observed in sponge [41]. These data indicate that the DNA fragmentation pathway in apoptosis is conserved in sponge. Thus an ancestral CIDE-N domain, the NCD of Dffa, should also exist in sponge. The exact conclusion awaits further sequencing information from sponges.

### The formation of CIDE-N and CIDE-C in early vertebrates

The mechanisms for the origin of new genes, such as exon shuffling, gene duplication and retroposition, have been thoroughly explored and extensively discussed [42]. It is well established that recombination of sequences encoding protein domains play a major part in protein evolution. However, there is less evidence to suggest how the novel protein domain, themselves, arise [43]. Here, we found homology sequences of CIDE-C exist only in vertebrates and all of them are linked to CIDE-N to form Cide. Based on protein sequence alignments (Fig 1, 2) and the phylogenetic tree (Fig 5a), CIDE-N domain of Cide family proteins must have been derived from the NCD of Dffa. In addition, from the exon boundaries derived from the protein sequence alignments results (Fig 1, 2), we could tell that exon 3 of *Cide *gene is derived from the exon 2 of *Dffa *gene. By comparison of the genic structures and intron phases between *Dffa *and *Cide *gene family, we found that the formation of CIDE-N domain had undergone three steps of intron changes from NCD of Dffa (See Fig 3a for relative intron positions). First, the phase-1 intron1 of *Dffa *was changed to the phase-0 intron 2 of *Cide*. Second, the phase-1 intron 2 of *Dffa *was changed to the phase-0 intron 3 of *Cide*. Third, the phase-2 intron1 of *Cide *was formed. These three intron changes underlie the evolution of the NCD of Dffa into CIDE-N, and provide insight into the molecular mechanisms regarding the origin of Cide family. Completion of the whole genome sequencing of sea lamprey, regarded as the most primitive vertebrate thus far, will help ultimately resolve the formation of CIDE-N and CIDE-C domain.

### The divergence of Cide family proteins in advanced vertebrates

The 2R hypothesis (two rounds of whole genome duplication in early vertebrate evolution) suggests that one round whole genome duplication happened at the root of the vertebrate lineage, followed by another round in Agnatha and Gnathostomata [44]. We found the evolution path of Cide proteins is in good agreement with the hypothesis. We propose that the appearance of ancient Cide family protein occurred at the root of the vertebrate lineage along with the first round of whole genome duplication. We suspect that the most ancient Cide family protein was most similar to Cideb, which then gave rise to the ancient Cidea/c around the Agnatha and Gnathostomata period which was probably accompanied with another round of whole genome duplicatoin. Cidec and Cidea were derived from ancient Cidea/c at the emerging of the actinopterygian fishes. In support of the above conclusions are the following observations: no Cide protein was found in amphioxus; only Cideb and Cidec are found in little skate and spiny dogfish; all of Cidea, Cideb and Cidec are found in zebrafish and *X. tropicalis*. According to the phylogenies of CIDE-N and CIDE-C domains (Fig 5) and the evolution of other gene families such as the Hox gene family in early vertebrates [45,46], we speculate that there should be one Cide protein in sea lamprey with strong resemblance to Cideb. The lack of evidence for the presence of any Cide protein in sea lamprey must be due to the incomplete genome database available.

We have also compared the tissue distributions of EST clones and experimental data (Table 2), and found that there are some differences in the expression patterns of Cide proteins between mammals and lower vertebrates, which surprisingly revealed an expression overlap between *Cideb *and *Cidec *in lower vertebrates but not in mammals. The coexpression of *Cideb *and *Cidec *in the liver, guts and WAT of lower vertebrates is in accordance with our above-mentioned evolutionary model for the early divergence of *Cideb *and *Cidec*. *Cidea *seems to be highly expressed in tissues unique to mammals, including BAT and mammary gland.

Although Cide family proteins were originally identified to induce cell death [2], many studies have also found they could play an important part in modulating energy homeostasis, aging and the development of metabolic diseases such as obesity and diabetes [20,21,24-26]. Considering the urgent need in evolution for vertebrates to modulate energy homeostasis and the emerging of warm blood animals, Cide family proteins may originally to function as "thrifty" genes and gradually evolve to be important regulator of metabolic pathways in mammals [47]. Combining the vertebrate origin and the control of metabolic pathways, Cide family proteins could be ideal targets for therapeutic intervention of metabolic diseases such as obesity and diabetes.

### Model for the evolutionary history of Cide family proteins

**Figure 6:**
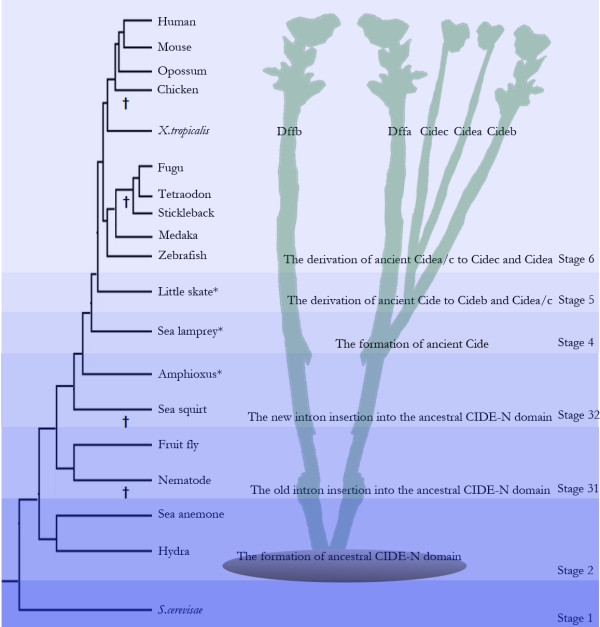
**The Evolutionary history of Cide family proteins based on a phylogenetic tree of animals**. The evolutionary history of Cide family proteins is divided into several stages based on the phylogenetic tree of animals [63–66]. '*' indicate three key model organisms whose whole genomes have not been fully sequenced thus far. Absence of Cide or Dff family proteins from each species is indicated by a cross next to the respective branch of the evolutionary tree. Model organisms used are *S. cerevisae*, hydra (*Hydra vulgaris*), sea anemone (*Nematostella vectensis*), nematode (*Caenorhabditis elegans*), fruit fly (*Drosophila melanogaster*), sea squirt (*Diazona violacea*), amphioxus (*Branchiostoma floridae*), sea lamprey (*Petromyzon marinus*), little skate (*Leucoraja erinacea*), zebrafish (*Danio rerio*), medaka (*Oryzias latipes*), stickleback (*Gasterosteus aculeatus*), tetraodon (*Tetraodon nigroviridis*), fugu (*Takifugu rubripes*), *X. tropicalis*, chicken (*Gallus gallus*), opossum (*Monodelphis domestica*), mouse (*Mus musculus*), human (*Homo sapiens*).

Based on our results, we mapped the presence or absence of the Cide and Dff family proteins to the phylogenetic tree of the animals, and summarized the evolutionary history of CIDE-N and CIDE-C domains into six stages (Fig 6). Around the transition of unicellular protozoan to multicellular metazoan, or the evolution of Bilateria from diploblasts (possibly the results of Cambrian explosion), one ancient or ancestral NCD for Dffa and Dffb was formed, encoded by an ancient exon bordering a phase 1 intron. Subsequent duplication led to the separation of Dffa and Dffb in cnidarians, and only the NCD of Dffa, but not Dffb, comprised the ancestral CIDE-N domain (Stage 2). In arthropods the ancient exon that encoded the ancestral CIDE-N domain was spliced by one phase 0 intron, while in nematodes the whole Dff family proteins, including the ancestral CIDE-N domain, were lost for some unknown reason (Stage 31). Around the same time in cephalochordates another phase 1 intron was inserted into a different position of the ancestral CIDE-N. This new intron insertion of the ancestral CIDE-N was later passed on to vertebrates. Also Dff family proteins might have disappeared from urochordates at this time (Stage 32). In early vertebrates like agnathan fishes, NCD of Dffa/the ancestral CIDE-N domain underwent duplication. One duplicated NCD of Dffa became the CIDE-N domain and merged with the newly formed CIDE-C domain to generate one ancient Cideb-like protein (Stage 4). Subsequent duplication led to the ancient Cidea/c protein which bears strong resemblance to Cidec in chondrichthyan fishes (Stage 5). When actinopterygian fishes occurred, Cidea was formed from the duplication of ancient Cidea/c. Some Cide family proteins might have disappeared in several vertebrate species (Stage 6).

## Conclusion

In this article, we searched various databases and performed comparative genomic analysis to study the sequence conservation, genomic structure, and phylogenetic tree of the CIDE-N and CIDE-C domains of Cide proteins. We were able to define signature sequences of CIDE-N domain and CIDE-C domain for Cide proteins, and NCD for Dff proteins, respectively. Our study identified the ancestral CIDE-N domain in cnidarians, and found the CIDE-C domain exists only in vertebrates. Further analysis of genomic structure such as exon length and intron phase patterns showed although evolution of the ancestral CIDE-N domain had undergone different intron insertions to various positions in the domain among invertebrates, the genomic structure of *Cide *family in vertebrates is stable with conserved intron phase. We propose that NCD of Dffa was duplicated in early vertebrates, and one of the duplicated copies became CIDE-N domain that merged with the newly formed CIDE-C domain, generating an ancient Cide family protein. Subsequent duplication and evolution led to the formation of different Cide family proteins that exert their specific roles in the control of metabolic pathways in different tissues.

## Methods

We retrieved human and mouse Cide and Dff family protein sequences using NCBI Entrez [48], their accession numbers are as follows: human Cidea (GenBank: AAQ65241) 219aa; human Cideb (GenBank: AAH35970) 219aa; human Cidec (GenBank: AAH16851) 238aa; human Dffa (GenBank: AAH07721) 331aa; human Dffb (GenBank: AAC39709) 338aa; mouse Cidea (GenBank: AAH96649) 217aa; mouse Cideb (GenBank: AAH12664) 219aa; mouse cidec/Fsp27 (Swiss-Prot: P56198) 239aa; mouse Dffa (GenBank: AAH58213) 331aa; mouse Dffb (GenBank: AAH53052) 343aa.

Using the MAFFT algorithm [49] implanted in Jalview [50], we performed alignments of the N-terminal regions of human and mouse Cide and Dff family proteins, and the CIDE-C domain of Cide proteins.

### Hmmer search of Nr.db from NCBI

Using the well-defined CIDE-N motif pfam02017, we searched potential Cide and Dff family proteins in the downloaded NCBI non-redundant protein database [48] by Hidden Markov model search program HMMER [51], and found 287 proteins with satisfying E cutoff(<10). Then we performed multiple sequence alignment analysis to identify the resultant proteins through Jalview [50]. The nonredundant entries were summarized in Table 1.

### tblastn search in EST database from NCBI

Using the two most conserved regions of mouse Cidea (a region in the CIDE-N domain of 37 amino acids: TLVLEEDGTVVDTEEFFQTLRDNTHFMILEKGQKWTP, and the other in the CIDE-C domain of the 35 amino acids: IARVTFDLYRLNPKDFLG CLNVKATMYEMYSVSYD) revealed by the multiple sequence alignment of human and mouse Cide proteins, we conducted two tblastn searches with the EST database from NCBI [52]. In order to analyze and compare the sequences we identified, we translated all of the cDNA sequences into protein sequences using the Translate Tool from the ExPaSy server [53]. Further sequence composition analysis and alignments were performed using Jalview [50].

We carried out sequence alignment for the N-terminals of Cide and Dff family proteins from three species: hydra, sea anemone, and human; we also conducted a full sequence alignment of human Cideb and a putative CIDE-N domain-containing protein in *C. elegans *using ClustalW [54]. By doing a pair wise comparison for each of the two proteins mentioned above using Vector NTI [55], we were able to determine the sequence homology between these proteins.

### Gene structure analysis using the genome database of 17 model organisms

Seventeen representative model organisms, including 11 vertebrates, 5 invertebrates and 1 fungus, were chosen in our gene structure analysis, as their genome sequences are either fully or mostly available. The genome for sea anemone was obtained from [56,57], amphioxus (*Branchiostoma floridae*) from [58], and for sea lamprey (*Petromyzon marinus*) from [59]. The genome databases for the other 14 organisms were obtained from Ensemble [60]. We summarized the nucleotide composition, length of the exons, and intron phase patterns bordering respective exons in tables, and genic structures to scale and exons to its translated protein regions in schematic figures.

### Phylogenetic analysis of Cide and Dff family proteins

We retrieved the sequences of 17 Cide family proteins in selected model vertebrates, and 14 Dff family proteins in selected vertebrates and invertebrates from their genome or EST databases (Additional file 1). By the preliminary multiple sequence alignments using the MAFFT algorithm, we isolated the CIDE-N and CIDE-C domain of Cide family proteins, and NCDs of Dff family proteins. After manual alignment improvement by Jalview (Additional file 2), the phylogeny of the 17 selected CIDE-N domains for Cide and the 14 NCD domains for Dff family proteins, the phylogeny of the CIDE-C domains and that of the CIDE-N domains are separately constructed by the neighbor-joining (NJ), maximum likelihood (ML), and unweighted pair group method with arithmetic mean (UPGMA) methods. We constructed NJ and UPGMA trees using MEGA 4.0 [61], and ML trees by using PHYML V2.4.4 [62]. For NJ and UPGMA trees, Poisson correction for amino acid sequences and 10,000 bootstrap resamplings were used, while the Jones, Taylor, and Thorton (JTT) model for amino acid sequences and 100 bootstrap resamplings were used in ML analysis. Tree files were viewed by using MEGA 4.0 [61]. NJ trees are shown with bootstrap values for NJ, ML and UPGMA analyses (first, second, and third values, respectively). Finally, we mapped the distribution of the Cide and Dff family proteins to the standard phylogenetic tree of the animals and summarized the evolutionary history of CIDE-N and CIDE-C domains into several stages.

## Authors' contributions

CW carried out the analysis of Cide and Dff family proteins in human and mouse, tblastn search in EST database from NCBI and helped to draft the manuscript. YZ carried out hmmer search in Nr.db from NCBI, tissue distribution analysis, genomic structure and phylogenetic analysis using the genome database of model organisms and drafted the manuscript. ZS participated in the hmmer search in Nr.db from NCBI. PL participated in experimental design, data coordination, analysis and interpretation. PL was also responsible for the revision, finalization of the manuscript and the decision to submit the manuscript for publication. All authors read and approved the final manuscript.

## Supplementary Material

Additional file 1 **Sequences of Cide and Dff family proteins used in our analysis**. This table summarizes Accession Numbers of the sequences used in our phylogenetic analysis. Click here for file

Additional file 2 **Multiple sequence alignments for each tree building in this study**. This figure depicts the multiple sequence alignments for each tree building in Fig 5 (In ALN format). Click here for file
