# Supplementary material for: Molecular evolution of Cide family proteins: Novel domain formation in early vertebrates and the subsequent divergence
Source: BMC Evol Biol. 2008 May 23;8:159. doi: 10.1186/1471-2148-8-159 (PMC2426694; doi:10.1186/1471-2148-8-159)
Supplement: Additional file 1 — Sequences of Cide and Dff family proteins used in our analysis. This table summarizes Accession Numbers of the sequences used in our phylogenetic analysis. [file 1471-2148-8-159-S1.pdf]

Sequences of Cide and Dff family proteins used in our analysis

Name	Accession number	Database
Human Cidea	gi|34396082|gb|AAQ65241.1|	NCBI
Mouse Cidea	gi|66794611|gb|AAH96649.1|	NCBI
Opossum Cidea	ENSMODP00000026771	Ensembl
Chicken Cidea	ENSGALP00000000023	Ensembl
X.tropicalis Cidea	gi|51513504|gb|AAH80498.1|	NCBI
Human Cideb	gi|23271172|gb|AAH35970.1|	NCBI
Mouse Cideb	gi|15215099|gb|AAH12664.1|	NCBI
Opossum Cideb	ENSMODP00000003715	Ensembl
X.tropicalis Cideb	gi|58332718|ref|NP_001011434.1|	NCBI
Shark Cideb	gi|56844740|gb|CX197316.1|	NCBI
Little skate Cideb	gi|51530603|gb|CV067338.1|	NCBI
Human Cidec	gi|16877166|gb|AAH16851.1|	NCBI
Mouse Cidec	gi|2829467|sp|P56198|	NCBI
Opossum Cidec	ENSMODP00000006434	Ensembl
X.tropicalis Cidec	gi|51703980|gb|AAH81304.1|	NCBI
Shark Cidec	gi|54674557|gb|CV652221.1|	NCBI
Little skate Cidec	gi|74508887|gb|DT726762.1|	NCBI
Human Dffa	gi|14043461|gb|AAH07721.1|	NCBI
Mouse Dffa	gi|34849796|gb|AAH58213.1|	NCBI
Opossum Dffa	ENSMODP00000003227	Ensembl
Chicken Dffa	gi|50759301|ref|XP_417610.1|	NCBI
X.tropicalis Dffa	gi|60416006|gb|AAH90808.1|	NCBI
Shark Dffa	gi|114788629|gb|EE885204.1|	NCBI
Little skate Dffa	gi|114865581|gb|EE992848.1|	NCBI
Amphioxus Dffa	estExt_fgenesh2_pg.C_3670025	Amphioxus Genome Database
Human Dffb	gi|3056727|gb|AAC39709.1|	NCBI
Mouse Dffb	gi|31892789|gb|AAH53052.1|	NCBI
Opossum Dffb	ENSMODP00000008058	Ensembl
Chicken Dffb	gi|17226274|gb|AAL37716.1|	NCBI
X.tropicalis Dffb	ENSXETP00000013293	Ensembl
Amphioxus Dffb	estExt_GenewiseH_1.C_8570010	Amphioxus Genome Database
