# Supplementary material for: Molecular evolution of Cide family proteins: Novel domain formation in early vertebrates and the subsequent divergence
Source: BMC Evol Biol. 2008 May 23;8:159. doi: 10.1186/1471-2148-8-159 (PMC2426694; doi:10.1186/1471-2148-8-159)
Supplement: Additional file 2 — Multiple sequence alignments for each tree building in this study. This figure depicts the multiple sequence alignments for each tree building in Fig 5 (In ALN format). [file 1471-2148-8-159-S2.pdf]

Multiple sequence alignments for each tree building in this study (In ALN format).

1. Multiple sequence alignments for Fig 5a.

```
Chicken Cidea                       RPFRVSNASRSSRKGIVASSLQELISKILEAFLITAGTVTLVLEEDGTVV
Human Cidea                         RPFRVSNHDRSSRRGVMASSLQELISKTLDALVIATGLVTLVLEEDGTVV
Mouse Cidea                         RPFRVSNHDRSSRRGVMASSLQELISKTLDVLVITTGLVTLVLEEDGTVV
Opossum Cidea                       RPFRVSNHDRSSRRGVTANTLKELINKTLDALAITTGLVTLVLEEDGTVV
X.tropicalis Cidea                  RPFRVSNSDRSSKKGIVAGTLKELIEKASETLFIHSDLVTLVLEEDGTVV
Human Cidec                         RPCRVSTADRSVRKGIMAYSLEDLLLKVRDTLMLADKPFFLVLEEDGTTV
Mouse Cidec                         RPCRVSTADRKVRKGIMAHSLEDLLNKVQDILKLKDKPFSLVLEEDGTIV
Opossum Cidec                       RPYRVSNADRSIRKGIMADSLLDLNHKVRGFRKVCGRHFSLVLEEDGTII
X.tropicalis Cidec                  RPFRVCNSNRSLRKGIVANSLEDLINKTQDALLM-LEAITLVLDEDGTCV
Little skate Cidec                  RPFRVCNWDRSLKKGIMALSLQDLLDKVQESLHT-TSSVSLLLEEDGTFI
Spiny dogfish Cidec                 RPFRVCNWDRSLKKGIMAESLRDLLDKVQESLHT-TCSISLLLEEDGTFI
Human Cideb                         RPFRVCDHKRTIRKGLTAATRQELLAKALETLLL-NGVLTLVLEEDGTAV
Mouse Cideb                         RPFRVCDHKRTVRKGLTAASLQELLDKVLETLLL-RGVLTLVLEEDGTAV
Opossum Cideb                       RPFRVCDHKRVTRKGLTAATRQELLDKALETLLL-SGVLTLVLEEDGTCV
X.tropicalis Cideb                  RPFRVCNHDRTVRRGVTAGSLRELIARAMDALFL-SGVVSLVLEDDGTQL
Spiny dogfish Cideb                 RPFRICNHDRTSRKGLTARTLGELINKAVDSLLL-TQVISVVLEEDGTAV
Little skate Cideb                  RPFRVCNHDRSRRSGVTAGSLRELIGKAMDAVLV-TGLVSLVLDEDGTLV
Chicken Dffa                        KPCVVRRGDGREQHGLAASSLRELRDKAGSALAIGGRPITLVLAEDGTIV
Human Dffa                          KPCLLRRNYSREQHGVAASCLEDLRSKACDILAIDLTPVTLVLAEDGTIV
Mouse Dffa                          KPCLLRRNHSRDQHGVAASSLEELRSKACELLAIDLTPITLVLAEDGTIV
Opossum Dffa                        KPCLLRRNRSREHHGAAASCLEDLKQKACDILEIDLEPITLVLAEDGTIV
X.tropicalis Dffa                   KACVVSLPGSKDRHGVAAASLEELLEKACKKFSLDVEPITLVLAEDGTIV
Litter skate Dffa                   RPFKVIGSSRKETFGVVAASLRELRDTGSKKLNLGQTHCTVVLEEDGTII
Spiny dogfish Dffa                  LPCRICDLGRLRTFGVVATSLTDLRNKGMEKLCLDKQTYTVVLEDDGTII
Amphioxus Dffa                      KAFRVWDGERKVKVGVVARSLHELKSKGRQHLNLPEEDLSIVLEEDGTVV
Chicken Dffb                        RGFRLRRPGSAQKFGAAAGSLRGLLRKGCRLLQLPLAGSRLCLYEDGTEL
Human Dffb                          KSVKLRALRSPRKFGVAGRSCQEVLRKGCLRFQLPERGSRLCLYEDGTEL
Mouse Dffb                          KCVKLRALHSACKFGVAARSCQELLRKGCVRFQLPMPGSRLCLYEDGTEV
Opossum Dffb                        RTFKLRALHSQDKYGVAGKSCQEVLLKGCQKFQLPISGSRLCLYEDGTEL
X.tropicalis Dffb                   RCYKIRTLHSQQKYGIACKNLAELKQKACRKFQLDCPTTCVCLYEDGTEV
Amphioxus Dffb                      KPFKIRSADDSQKYGVAAQDLNDLIAKGCKVLKVPIKGCKICLQQDGTLI
                                        :         *        :                : * :*** :

Chicken Cidea                       DTEEFFQSLNDNTHFMVLEKGQKWTQ
Human Cidea                         DTEEFFQTLGDNTHFMILEKGQKWMP
Mouse Cidea                         DTEEFFQTLRDNTHFMILEKGQKWTP
Opossum Cidea                       DTEEFFQTLGDNTHFMILEKGQKWTL
X.tropicalis Cidea                  DTEDFFQSLEDNTQFLLLEAKQKWTQ
Human Cidec                         ETEEYFQALAGDTVFMVLQKGQKWQP
Mouse Cidec                         ETEEYFQALAKDTMFMVLLKGQKWKP
Opossum Cidec                       ETEEYFQTLNEDTVFMVLQKGQKWQP
X.tropicalis Cidec                  DTEEFFRSLDDGAVFMALAKGQKWKP
Little skate Cidec                  ESEEFFQTVLDHTLFMVIEKGQKWTL
Spiny dogfish Cidec                 DTEEFFQTVLDNTLFMVLEKGQKWTL
Human Cideb                         DSEDFFQLLEDDTCLMVLQSGQSWSP
Mouse Cideb                         DSEDFFQLLEDDTCLMVLEQGQSWSP
Opossum Cideb                       ESEEFFQMLEDDTSLMVLEAGQNWSP
X.tropicalis Cideb                  DREDFFETLEDGSVVMVLEKGQKWMP
Spiny dogfish Cideb                 EGEDFFEHLDEDTTLMVLEKGQRWTS
Little skate Cideb                  DTEDFFRHLEDNTRLMVLEKGQSWRS
Chicken Dffa                        DDEDYFLCLPSNTKFVALAEGERWSG
Human Dffa                          DDDDYFLCLPSNTKFVALASNEKWAY
Mouse Dffa                          DDDDYFLCLPSNTKFVALACNEKWTY
Opossum Dffa                        DDEDYFLCLPSNTKFVALTCNEKWTY
X.tropicalis Dffa                   EDEDYFLCLPPNTKFVILTGNKKWAP
Litter skate Dffa                   DDEKYFAHMPKDTKFMILGAGEKWVP
Spiny dogfish Dffa                  DDEDYFMHLPENTKFMILGSSE----
Amphioxus Dffa                      ADNEFFQFIPDQTVVQLLTEGQEWRP
Chicken Dffb                        -SEAFFRTLPPQTELVLLRPGESWPG
Human Dffb                          -TEDYFPSVPDNAELVLLTLGQAWQG
Mouse Dffb                          -TDDCFPGLPNDAELLLLTAGETWHG
Opossum Dffb                        -TEGYFQSIPDNTELMLLTPGQSWQG
X.tropicalis Dffb                   -SEEYLETVPDNTVLLLLTPGQTWQG
Amphioxus Dffb                      NSREFFQALPPLSVLVFLRKGEKWTG
                                         :  :   : .  :   :
```

2. Multiple sequence alignments for Fig 5b.

```
Chicken Cidea                       RQKKKMGVANITFDLYKLNPKDFIGCLNVKATFYEIYSVSYDIKCMGAKS
Human Cidea                         SPPKRSGIARVTFDLYRLNPKDFIGCLNVKATMYEMYSVSYDIRCTGLKG
Mouse Cidea                         KQPKKSGIARVTFDLYRLNPKDFLGCLNVKATMYEMYSVSYDIRCTRFKA
Opossum Cidea                       QKQKKSGIARVTFDLYKTNPKDFLGCLNVKATMYEMYSVSYDIRCTSAKA
X.tropicalis Cidea                  QHEKKTGIANLTFDLYKLNPKDFGGCLNIKATFYEIYSISWDMQFLGAKK
Human Cidec                         KPAKKIDVARVTFDLYKLNPQDFIGCLNVKATFYDTYSLSYDLHCCGAKR
```

```
Mouse Cidec                    KPTKKIDVARVTFDLYKLNPQDFIGCLNVKATLYDTYSLSYDLHCYKAKR
Opossum Cidec                  KPSKKVDVASVTFDLYKARPDDFIGCLNVKATLYGNYTLSYDLHCYGAKR
X.tropicalis Cidec             KPARKIDVACVSFDLYKNHPRDFIGCLNVKATLYGTYSLSYDLQCYGAKR
Little skate Cidec             KLCRR-DVARITFDLYKESPQDFIGCLNVKVTLYGSYSLSCDVQCTGAKK
Spiny dogfish Cidec            KLRRRKDVARITFDLYKENPQDFIGCLNVKVTLYGSYSLSYDVQCVGAKK
Human Cideb                    RPKHSKDIARFTFDVYKQNPRDLFGSLNVKATFYGLYSMSCDFQGLGPKK
Mouse Cideb                    KPKHSKDIARITFDVYKQNPRDLFGSLNVKATFYGLYSMSCDFQGVGPKR
Opossum Cideb                  KPKHSKDIASITFDVYKQSPRDLFGSLNVKATFYGLYSMSCDFQGLGPKK
X.tropicalis Cideb             KPRNSKDIAKVTFNVYKLNPRDLFGSLNIKATFYGLYSMSCDFQCLGPKK
Spiny dogfish Cideb            KPKNSKDIARITFDIYKLHPKDIFGSLNVRATFYGLYSMTFDIKCLGPKK
Little skate Cideb             KPKNSKDIARITLDIYKRNPRDLF--------------------------
                                  .  .:* .::::*:  * *:

Chicken Cidea                  ILRKVLQIISHVAQITGQFLLYTGTY
Human Cidea                    LLRSLLRFLSYSAQVTGQFLIYLGTY
Mouse Cidea                    VLRNLLRFMSYAAQMTGQFLVYAGTY
Opossum Cidea                  VLRQLLRIMSYAAQITGQFLVYGGTY
X.tropicalis Cidea             VLRQLIRGLSYLAQVTGHLLLYGGSF
Human Cidec                    IMKEAFRWALFSMQATGHVLLGTSCY
Mouse Cidec                    IVKEMLRWTLFSMQATGHMLLGTSSY
Opossum Cidec                  IMKEALRCALFSMQATGHILLGTSYF
X.tropicalis Cidec             MVKEALRWTLYTMQATGHVLLGTSCY
Little skate Cidec             IMREALRLTMYTMQATGHILLG----
Spiny dogfish Cidec            IMREALRLTMFTMQATGHILLGTSCY
Human Cideb                    VLRELLRWTSTLLQGLGHMLLGISST
Mouse Cideb                    VLRELLRGTSSQLQGLGHMLLGISST
Opossum Cideb                  VLRSLLRWTSRLLQGLGNMLLSVSST
X.tropicalis Cideb             VLREFLKFLSVTLQGIGRVLLTASGV
Spiny dogfish Cideb            VIRELLRVISSLMYAVGQTLVTGSSL
Little skate Cideb             --------------------------
```

3. Multiple sequence alignments for Fig 5c.

```
Chicken Cidea                  RPFRVSNASRSSRKGIVASSLQELISKILEAFLITAGTVTLVLEEDGTVV
Human Cidea                    RPFRVSNHDRSSRRGVMASSLQELISKTLDALVIATGLVTLVLEEDGTVV
Mouse Cidea                    RPFRVSNHDRSSRRGVMASSLQELISKTLDVLVITTGLVTLVLEEDGTVV
Opossum Cidea                  RPFRVSNHDRSSRRGVTANTLKELINKTLDALAITTGLVTLVLEEDGTVV
X.tropicalis Cidea             RPFRVSNSDRSSKKGIVAGTLKELIEKASETLFIHSDLVTLVLEEDGTVV
Human Cidec                    RPCRVSTADRSVRKGIMAYSLEDLLLKVRDTLMLADKPFFLVLEEDGTTV
Mouse Cidec                    RPCRVSTADRKVRKGIMAHSLEDLLNKVQDILKLKDKPFSLVLEEDGTIV
Opossum Cidec                  RPYRVSNADRSIRKGIMADSLLDLNHKVRGFRKVCGRHFSLVLEEDGTII
X.tropicalis Cidec             RPFRVCNSNRSLRKGIVANSLEDLINKTQDALLM-LEAITLVLDEDGTCV
Little skate Cidec             RPFRVCNWDRSLKKGIMALSLQDLLDKVQESLHT-TSSVSLLLEEDGTFI
Spiny dogfish Cidec            RPFRVCNWDRSLKKGIMAESLRDLLDKVQESLHT-TCSISLLLEEDGTFI
Human Cideb                    RPFRVCDHKRTIRKGLTAATRQELLAKALETLLL-NGVLTLVLEEDGTAV
Mouse Cideb                    RPFRVCDHKRTVRKGLTAASLQELLDKVLETLLL-RGVLTLVLEEDGTAV
Opossum Cideb                  RPFRVCDHKRVTRKGLTAATRQELLDKALETLLL-SGVLTLVLEEDGTCV
X.tropicalis Cideb             RPFRVCNHDRTVRRGVTAGSLRELIARAMDALFL-SGVVSLVLEDDGTQL
Spiny dogfish Cideb            RPFRICNHDRTSRKGLTARTLGELINKAVDSLLL-TQVISVVLEEDGTAV
Little skate Cideb             RPFRVCNHDRSRRSGVTAGSLRELIGKAMDAVLV-TGLVSLVLDEDGTLV
Amphioxus Dffa                 KAFRVWDGERKVKVGVVARSLHELKSKGRQHLNLPEEDLSIVLEEDGTVV
                               :. *:   .*  : *: * :  :*  :           . ::*::*** :

Chicken Cidea                  DTEEFFQSLNDNTHFMVLEKGQKWTQ
Human Cidea                    DTEEFFQTLGDNTHFMILEKGQKWMP
Mouse Cidea                    DTEEFFQTLRDNTHFMILEKGQKWTP
Opossum Cidea                  DTEEFFQTLGDNTHFMILEKGQKWTL
X.tropicalis Cidea             DTEDFFQSLEDNTQFLLLEAKQKWTQ
Human Cidec                    ETEEYFQALAGDTVFMVLQKGQKWQP
Mouse Cidec                    ETEEYFQALAKDTMFMVLLKGQKWKP
Opossum Cidec                  ETEEYFQTLNEDTVFMVLQKGQKWQP
X.tropicalis Cidec             DTEEFFRSLDDGAVFMALAKGQKWKP
Little skate Cidec             ESEEFFQTVLDHTLFMVIEKGQKWTL
Spiny dogfish Cidec            DTEEFFQTVLDNTLFMVLEKGQKWTL
Human Cideb                    DSEDFFQLLEDDTCLMVLQSGQSWSP
Mouse Cideb                    DSEDFFQLLEDDTCLMVLEQGQSWSP
Opossum Cideb                  ESEEFFQMLEDDTSLMVLEAGQNWSP
X.tropicalis Cideb             DREDFFETLEDGSVVMVLEKGQKWMP
Spiny dogfish Cideb            EGEDFFEHLDEDTTLMVLEKGQRWTS
Little skate Cideb             DTEDFFRHLEDNTRLMVLEKGQSWRS
Amphioxus Dffa                 ADNEFFQFIPDQTVVQLLTEGQEWRP
                                 :::*. :   : .  :   * *
```
